# Discover protein sequence signatures from protein-protein interaction data

**DOI:** 10.1186/1471-2105-6-277

**Published:** 2005-11-23

**Authors:** Jianwen Fang, Ryan J Haasl, Yinghua Dong, Gerald H Lushington

**Affiliations:** 1 Bioinformatics Core Facility, University of Kansas, Lawrence, KS 66045, USA; 2 Information and Telecommunication Technology Center, University of Kansas, Lawrence, KS 66045, USA; 3 Molecular Graphics and Modeling Laboratory, University of Kansas, Lawrence, KS 66045, USA

## Abstract

**Background:**

The development of high-throughput technologies such as yeast two-hybrid systems and mass spectrometry technologies has made it possible to generate large protein-protein interaction (PPI) datasets. Mining these datasets for underlying biological knowledge has, however, remained a challenge.

**Results:**

A total of 3108 sequence signatures were found, each of which was shared by a set of guest proteins interacting with one of 944 host proteins in *Saccharomyces cerevisiae *genome. Approximately 94% of these sequence signatures matched entries in InterPro member databases. We identified 84 distinct sequence signatures from the remaining 172 unknown signatures. The signature sharing information was then applied in predicting sub-cellular localization of yeast proteins and the novel signatures were used in identifying possible interacting sites.

**Conclusion:**

We reported a method of PPI data mining that facilitated the discovery of novel sequence signatures using a large PPI dataset from *S. cerevisiae *genome as input. The fact that 94% of discovered signatures were known validated the ability of the approach to identify large numbers of signatures from PPI data. The significance of these discovered signatures was demonstrated by their application in predicting sub-cellular localizations and identifying potential interaction binding sites of yeast proteins.

## Background

The development of high-throughput technologies for discovering interactions between proteins has made it possible to screen entire proteomes and produce large protein-protein interaction (PPI) datasets. Different methods of PPI detection, including yeast two-hybrid assays [1-3], mass spectrometry of coimmunoprecipitated protein complexes [4,5], and correlated messenger RNA profiles [6,7], discover PPIs of variable reliability and the majority of putative PPIs are of low confidence. Despite the presence of false positives, the wealth of PPI data generated over the past several years is the source of many publicly available databases, such as the Database of Interacting Proteins (DIP [8]) and the MIPS mammalian protein-protein interaction [9]. The availability of these large datasets is now enabling researchers to predict undiscovered PPIs and hypothesize the function and sub-cellular localization of proteins.

**Figure 1:**
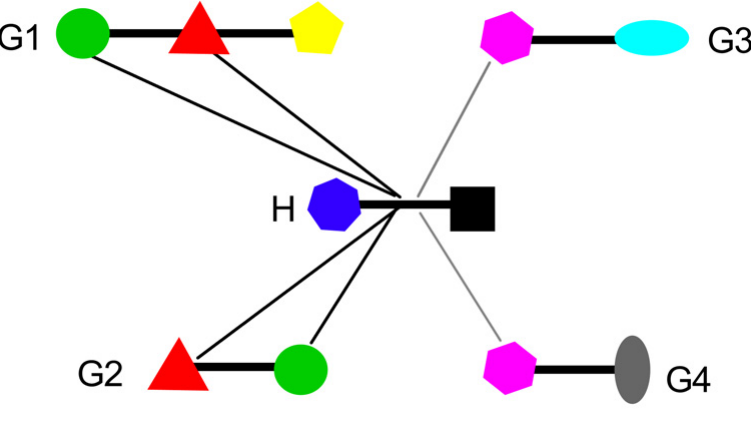
A scheme illustrates the procedure of inferring DDIs from PPIs. Colored shapes represent sequence signatures. Suppose protein H (the host) interacts with four guest proteins (G1, G2, G3, G4) and all signatures in the schema are known with the exception of the one represented by purple hexagon. In this case only interactions with G1 and G2 are useful in inferring DDIs. In this study we used MEME program to identify all signatures shared by guests.

PPI data has been used to analyse domain-domain interactions (DDIs), based upon the widely accepted hypothesis that proteins interact with one another via conserved domains (Figure 1). Large-scale PPI databases are used to identify correlated domains that are implicated in the binding of protein partners. When one of these sequence signatures is observed in a newly discovered protein, it is possible to predict its interactions with other proteins based on the knowledge base of correlated domains. DDIs were thus used to predict the function and PPIs of newly discovered proteins [10]. Deng et al. [11] used maximum likelihood estimation to discover DDIs, which were then used to predict the likelihood of interaction for any protein pair. Other recent forms of DDI analysis include the use of interacting domain profile pairs [12], and a domain combination based probabilistic framework [13].

Very recently, PPI data, in conjunction with structural information, were used to produce a set of putative binding motif pairs [14]. The significance of motif discovery stems from the idea that the actual binding sites most directly responsible for the binding of proteins are probably smaller than whole domains. Thus, the discovery of these smaller sequence signatures allows researchers to structurally characterize PPIs with more precision.

This study was also based on the assumption that PPIs result from the interactions of conserved sequence signatures. Unlike Li and Li's work [14], our method of PPI data mining did not use structural data, which are well known to be biased towards small, globular proteins. In this paper, a set of guest proteins represents those proteins known from PPI database to share a common interacting partner, i.e. a host protein. If a protein interacts with itself, it is a host as well as a guest. Signatures shared by sets of guest proteins were initially discovered using the program MEME [15] on a large PPI dataset. Searches of sequence signature databases for the identified motifs revealed that 84 distinct motifs had not been characterized previously. The significance of these newly discovered signatures was then demonstrated by their application in predicting the sub-cellular localization of yeast proteins and identifying potential interacting sites.

## Results

A sequence signature is defined as a "highly conserved region", a sequence pattern that is found repeatedly in a group of related protein sequences [15]. By this definition, a sequence signature could be a protein family, functional domain, functional site, or any conserved region of unknown function, and thus the actual physical manifestation of a signature can vary greatly in size. In our study, sequence signatures were derived from MEME motifs. We wrote numerous Perl scripts and used a MySQL relational database to facilitate the processes of data collection, program execution, and data analysis.

### Discovery of sequence signatures

The 1923 batch executions of MEME yielded 3108 sequence signature models shared by the 1555 distinct guest proteins of 944 host proteins from *Saccharomyces Cerevisiae *(baker's yeast) (see details in Methods). Of the 6770 distinct PPIs actually involved in building these signature models, 1509 (22.3%) were identified as high confidence interactions in the PPI dataset. When compared to the percentage of high confidence PPIs in the input files (20.7%), the percentage of high confidence PPIs used to construct motif models represents a statistically significant difference (*p*-value = 0.0013, two-tailed t-test).

Signature model length varied from 10 to 300 residues: the minimum and maximum lengths specified for each MEME execution. Only 25 models (<1%) were as long as 300 residues, which indicated that the maximum length used in this study was appropriate. MEME splits one sequence signature in two if its length is greater than the specified maximum. Thus, less than 2% of the 3108 models were the result of splitting sequence signatures. The average model length was 33.6 residues, with a standard deviation of 40.3. It should be pointed out that there was redundancy among these signatures because different host proteins may interact with similar sets of guest proteins. We did not attempt to identify distinct signatures because that was not the main goal of the present study. Instead, we identified distinct novel sequence signatures (84 distinct signatures out of 172 initial results, see below for details). Thus, we estimated that overall about half of these signatures were distinct.

### Occurrence of discovered sequence signatures in the yeast genome

Using the 3108 signature models discovered in MEME as input, MAST [16] was used to scan the entire genome of *S. cerevisiae *for occurrences of these sequence signatures in proteins that were not used to build the motif models. 1,993 protein sequences contained one or more of the sequence signatures, a 28% increase over the 1,555 proteins used to construct the signatures. Although this increase indicates that the newly discovered sequence signatures have some potential predictive value, any predictions based on these sequence signatures would be limited to approximately one-third of the *S. cerevisiae *genome. A broader application will be feasible only when more reliable PPI data are available.

### Novelty of discovered sequence signatures

Using the standalone version of InterProScan, the consensus sequences of 2337 of the discovered motif models were found to match signatures listed in one of the InterPro member databases. When the online version of InterProScan was used, an additional 599 sequence signatures were matched to un-integrated entries of the InterPro member databases. 172 novel sequence signatures remained. FASTA searches, which were the basis for the grouping of similar/identical sequence signatures, resulted in the creation of 84 distinct, novel sequence signatures. The length of these novel sequence signatures ranged from 10 to 36 residues. Table 1 provides a list of several of these novel signatures. A complete list can be found on the supplementary website . Interestingly, when InterProScan was used to match consensus sequence signatures to the Pfam database alone, only 545 (~18%) of the signatures were matched to known signatures.

**Table 1:** Novel sequence signature examples.

**Signature id**	**Host**	**Consensus sequence**	**Length**
YDL166C_1	YDL166C	EVLCCQLPKWCGFFQM	16
YML094_4	YML094	QRQGKLEVPGYVDIVKTSSGNEMPPQ	26
YOL094C_3	YOL094C	LWVEKYRPKNLDEVCGN	17
YGL063W_2	YGL063W	VKAVEGRKKGKEGKASQLVDLKFALAEDKV	30
YOR335C_5	YOR335C	AQSVGCRVDFKNPHDIIEGINAGEIE	26

### Localization prediction

Using signature sharing information, the sub-cellular localizations of 108 proteins were predicted based on the known locations of 5416 budding yeast proteins (see details in Methods). 52 predictions agreed with the ontology annotations of the SGD and 24 disagreed (~68% accuracy). The accuracy of the remaining 32 (Table 2) predictions could not be assessed, as the locations of these proteins have yet to be determined empirically. It is reasonable to believe these predictions would have similar prediction accuracy.

**Table 2:** Predicted localizations without known annotations from SGD. Evidence notation: 1: the ORF is a host, all or most guests are in the same location. 2: a guest, its host and all or most siblings are in the same location; 3: also a guest, but the location of host is unknown, all or most siblings are in the location. If there are multiple predictions for one ORF, the evidence and/or host names are concatenated in the corresponding columns.

**ID**	**ORF**	**Predicted_location**	**Evidence(s)**	**Host name(s)**
1	Q0105	cytoplasm	1	
2	YAL046C	cytoplasm, nucleus	1	
3	YAR073W	cytoplasm	2	YMR217W
4	YBL041W	cytoplasm, nucleus	1,2	YJL001W, YPR103W, YGR135W, YML092C, YGR253C, YER094C, YGL011C
5	YBL092W	cytoplasm, nucleus	2	YGR034W, YDL191W
6	YBR257W	cytoplasm, nucleus	2	YHR203C, YJR014W, YJR145C
7	YCR031C	cytoplasm, nucleus	2	YGR034W
8	YCR072C	cytoplasm, nucleus	1	
9	YDL075W	cytoplasm, nucleus	3	YDR292C
10	YDR064W	cytoplasm, nucleus	1,2	YGR262C, YAL035W
11	YDR109C	cytoplasm, nucleus	2	YJR024C
12	YDR287W	cytoplasm, nucleus	2	YEL041W
13	YEL041W	cytoplasm, nucleus	1,2	YDL236W, YHL046C
14	YER094C	cytoplasm, nucleus	1,2,3	YFR050C, YGL011C, YPR103W, YBL041W, YJL001W, YML092C, YGR253C, YGR135W
15	YGL063W	cytoplasm, nucleus	1,2	YDR158W, YDR007W
16	YGL224C	cytoplasm, nucleus	2	YMR009W, YDL219W, YJR024C
17	YHR016C	cytoplasm, actin	2	YBL007C
18	YHR044C	cytoplasm, nucleus	2	YDR074W
19	YJL213W	cytoplasm	2	YGR094W
20	YKL104C	cytoplasm, nucleus	2	YDR127W, YPL160W, YDR211W, YDR394W, YER110C
21	YLR209C	nucleolus, nucleus	1	
22	YLR359W	cytoplasm	2	YGL234W
23	YMR084W	cytoplasm, nucleus	1,2	YDR211W
24	YMR130W	cytoplasm, nucleus	2	YJR024C
25	YMR217W	cytoplasm	1	
26	YOL114C	cytoplasm, nucleus	2	YPL160W
27	YOR054C	cytoplasm, nucleus	2	YDR454C, YBR252W
28	YOR093C	cytoplasm	2	YBR208C
29	YOR111W	actin	2	YDL161W
30	YPL003W	cytoplasm, nucleus	2	YDR054C
31	YPL171C	cytoplasm, nucleus	2	YKR031C
32	YPL217C	nucleolus,nucleus	2	YLR197W, YHR052W, YDR449C

### Homology modeling and detection of putative interacting sites

The exact biological meanings of these novel sequence signatures can only be determined by web-lab experiments. One possible role of these signatures is to serve as the binding sites for protein-protein interactions. A binding site should have significant exposure to solvent. In order to assess this possibility, we built homology models for those yeast proteins containing novel signatures and having good model templates [see Additional file 1]. Using DSSP software program [17], we calculated the proposition of residues of signatures appearing on the surface (residues with solvent exposed surface ≥ 25 Å^2^). Statistical analysis (two-sided Fisher's exact test) confirmed that residues of signatures occurred on the surface more frequently than would be expected by chance (P < 0.04, Fisher's exact test). Thus we hypothesized these signatures are potential binding sites and plan to use site-directed mutagenesis and NMR spectrometry to verify the bioinformatics results.

## Discussion

Although independent, the PPI data mining method presented here is similar to that proposed by Li and Li [14]. Their research focused on motif pairs located on protein surfaces, and motif discovery was, in part, based on three-dimensional structures of proteins. Our method did not rely on PDB structural information, which is known to be biased towards small, globular proteins. Even without the additional structural information, many of the novel sequence signatures discovered in this study appear in the surfaces of proteins. Thus they are likely interacting sites.

Approximately 94% of the sequence signatures discovered in this study matched known sequence patterns, confirming the ability of this method to discover sequence signatures involved in various biological functions. It is our contention that the 84 novel sequence signatures reported in this study likely play biological roles such as interacting sites, and we are planning wet-lab experiments to investigate their functions.

The lengths of the novel sequence signatures are quite short, ranging from 10 to 36 residues. This is not surprising, as the yeast genome has been the subject of a remarkable number of studies and the majority of long sequence signatures are likely already known. Additionally, longer sequence signatures tend to contain gaps, and will thus be interpreted as multiple shorter signatures by MEME. Nevertheless, the discovery of short, novel sequence signatures, based on medium- and high-confidence PPIs, suggests that short sequence signatures do play biologically significant roles.

Only 545 (~18%) of the discovered sequence signatures matched known signatures in Pfam: a significantly smaller number than the 2936 signatures matched to one or more InterPro member databases. This result highlights a potential shortcoming of PPI predictions based on the analysis of DDIs inferred from Pfam data alone (e.g. ref [11]). The use of a single domain databases, such as pfam database with the average length of 145 amino acids [18] might cause a researcher to miss many important short sequence signatures, thereby decreasing prediction accuracy.

The use of PPI data to predict the sub-cellular localization of proteins is based on an intuitively simple idea: proteins that are found in the same location within a cell are more likely to interact with one another than proteins that are not. Ten subcellular compartments were actually used in our study. The resulting accuracy of PPI-based prediction of sub-cellular localization is reasonably good in this study and, at ~68%, represents a substantial increase in accuracy relative to what would be achieved (37%) if cytoplasm, the most populated compartment, was predicted for all systems. Our accuracy is comparable to that achieved in other recent studies. For example, using a hybrid system of gene ontology, functional domain and pseudo amino acid composition approaches, Chou and Cai obtained 70% of overall success identification rate [19,20]. Our accuracy rate was inferior to others that used fewer localization categories (for example, 88% accuracy rate based on cross validation was achieved when only four localization categories were used in ref [21]), but it is perfectly natural that a more ambitious categorization scheme such as ours should have a greater margin of error. Also we should emphasize that our approach represents a very intuitive and simple scheme based on PPI induced sequence signatures alone, in contrast to complicated hybrid systems employed in previous studies. Admittedly, our approach can only be used in predicting the localization of proteins involving in currently known PPIs, thus a broader application will be feasible only when more PPI data are available.

One of the major challenges to mining PPI data is the presence of numerous false positives, resulting from the deficiencies of current high-throughput screening techniques. The PPI data produced by some screening techniques such as yeast two-hybrid systems has been estimated to contain as much as 91% false interactions [22]. The 11,161 PPIs used as input to MEME were identified as medium or high confidence interactions, of which 20.7 % were high confidence. Of the PPIs actually used to build sequence signatures, 22.3% were high confidence interactions, a statistically significant increase of 7.7% over the original dataset. The disproportionate use of high-confidence PPIs to build sequence signatures supports the validity of the original reliability assignments, and suggests a method by which one may increase confidence in putative PPIs. Nevertheless, the quality of the results generated by all forms of PPI data mining remains constrained by the quantity and quality of the PPI data currently available. Consequently, the reliability of predictions based on PPI data is expected to increase as PPI databases increase in accuracy, size and taxonomic range.

## Conclusion

In conclusion, we have reported a novel procedure by which sequence signatures were discovered based on a large PPI dataset from *Saccharomyces cerevisiae*. The majority of these sequence signatures were matched with known sequence signatures present in the InterPro member databases. Nevertheless, 84 distinct sequence signatures were novel, and may be involved in the interactions of the proteins containing them. The sub-cellular localizations of 108 proteins of the yeast genome were predicted, based on the known locations of other proteins and PPI dataset. Of the 108 localization predictions, 52 agreed with SGD annotations, and 24 disagreed. The localization of remaining 32 proteins was experimentally unknown. However, it is reasonable to believe these predictions would have similar prediction accuracy.

Wet-lab experiments to determine the biological function of the discovered novel sequence signatures are being planned. We are also in the process of developing an algorithm that will enable the discovery of gap-containing sequence signatures based on PPI data. The PPI data mining method presented here is imminently applicable to other genomes associated with large PPI datasets. For example, we conducted similar study on the *E. Coli *genome and were able to identify 22 novel signatures (the results of which can be found in the complementary website).

## Methods

### Dataset

PPI data specific to the genome of *Saccharomyces Cerevisiae *(baker's yeast) were used because the quantity of PPI data available for yeast exceeds that of any other model organism. The ~6000 proteins of the yeast proteome could potentially produce more than 18 million distinct, guest-host interactions, though the actual number of PPIs is certainly much smaller, probably less than 100,000 [23,24]. However, PPIs are dynamic, and the empirical discovery of these interactions is time and location dependent. The current list of putative PPIs between proteins of the yeast proteome, therefore, does not represent all PPIs that occur in the cells of yeast.

The dataset used here was reported by von Mering et al. [23]. It contained 78380 non-redundant PPIs from yeast, which were assigned to three categories of reliability: 2455 high confidence, 9400 medium confidence, and 66535 low confidence. PPIs of this dataset were discovered by various experimental and computational methods including yeast two-hybrid systems, mass spectrometry technologies.

In an attempt to minimize the occurrence of false positives, only those PPIs assigned a reliability of high or medium confidence were used (2617 host proteins involved in 11855 interactions). Because MEME requires input in the form of set of two or more related proteins, 694 host proteins that interacted with only one protein were also excluded. Of the remaining 1923 host proteins, only 25 were involved in more than 100 distinct PPIs, including the most interactive protein, YPR110C, which was involved in 118 putative PPIs.

### MEME and MAST

MEME (v.3.0.10) was used to search for signatures shared by each group of guest proteins. MEME implements an unsupervised learning algorithm and ultimately produces one or more probabilistic signature models based on this input. The statistical significance of each signature model is quantified as an expectation value (E-value), which is an estimate of the number of signatures that would possess a higher log-likelihood ratio given randomly-generated training sequences. All signatures discovered by MEME are gapless, and the best width, number of occurrences, and description of each motif are based on statistical models.

For each of the 1923 host proteins associated with two or more guest proteins, a multiple sequence FASTA file was created from the amino acid sequences of its guest proteins. In every instance, MEME was executed with the following options: a minimum motif width of 10, maximum motif width of 300, maximum E-value of 0.1, and 5 as the maximum number of motifs.

MEME output files were then used as input for MAST (v3.0). MAST was used to search the entire yeast proteome for the sequence signatures described in the MEME output files. MAST output consists of the sequence name of each high-scoring match as well as the E-value of each match. For all MAST executions, the maximum E-value was set to 0.1. The results of MAST searches were used to assess the sequence coverage of sequence signatures identified by MEME and the usefulness of MEME output to PPI prediction.

### Signature model comparison

InterPro [25] is an integrated collection of the most commonly used databases of protein families, domains, and functional sites. The program InterProScan allows a user to search for sequence signatures in any number of these databases simultaneously [26]. Only LAMA can be used to compare MEME results to the BLOCKS database [27], but no tools currently exist for comparison to other sequence signature databases. Therefore, the consensus sequence of each motif model identified by MEME was searched for in all InterPro member databases, using the standalone version of InterProScan (release 4.0) and a local copy of InterPro (release 8.1). Signatures that were unsuccessfully matched with any entries in the local InterPro database were input to the online version of InterProScan to identify matches to known signatures that were not integrated into the InterPro database (i.e., thus unavailable in the local database). Those signatures that remained unmatched were considered novel. Because different host proteins may share the same set of guest proteins, some of these novel signatures were identical or similar. Thus, FASTA [28] searches were performed, using each potentially novel signature as a query sequence, and the set of all potentially novel signatures as a local database. We tested several E-values (0.1, 0.5, 1) and found that 0.5 was the best for distinguishing signatures. Higher threshold E-values led to the identification of signature pairs as similar when only one or two contiguous residues were identical, while lower values excluded the detection of signatures that were clearly similar. To compare the coverage of the individual InterPro member databases, each consensus sequence signature was also assessed using the Pfam database only.

Querying sequence signature databases with the consensus sequence of a MEME model, rather than the model itself, is similar to the approach proposed by Kahsay et al. [29], which facilitated the comparison of two Hidden Markov Models. To verify the appropriateness of using consensus sequences in lieu of the actual models, we queried the consensus sequences of several signature models along with each of their component sequences against InterPro databases. We found the hits of the consensus sequences were consistent to those of their component sequences. For example, the consensus sequence of the signature YPL049_1 matched to all significant signatures that two component sequences had. The only difference was that two residues of the consensus sequence additionally matched to an un-integrated signature. This match was insignificant considering that the length of the signature was 65 residues.

### Prediction of protein subcellular localization

Two proteins that interact with one another are likely found in the same subcellular location [23]. Thus PPI data can be used to predict the subcellular localizations of proteins. However, PPI data alone are currently not sufficient to predict subcellular localization due to the generally low reliability of current PPI data. In this study, we added an additional layer of confidence to predictions of subcellular localization by including our knowledge of sequence signatures shared by the guests of a host protein. For a guest protein with unknown localization, if its host protein and at least half of its fellow guest proteins shared a subcellular location, that guest protein was predicted to share this location as well. Similarly, if the localization of a host protein was unknown, and more than half of its guest proteins shared a common subcellular localization and one or more sequence signatures, the host protein was predicted to exist in the localization (Table 3).

**Table 3:** An example of protein location prediction. The host YGL115W has four guest proteins that share four statistically significant signatures. The host and all its guests with known location were found in cytoplasm. Thus the location of YGL208W was predicted as cytoplasm. The prediction was then confirmed with the ontology annotation in SGD database. The *p*-value of the occurrence is the probability that a single random subsequence of the length of the motif matches the motif.

**Guest**	**Motif ID**	***P*-value**	**Guest location**
YER027C	YGL115W_1	3.17E-76	cytoplasm
YGL208W	YGL115W_1	7.48E-75	
YDR422C	YGL115W_1	4.78E-48	cytoplasm
YER027C	YGL115W_2	3.87E-56	cytoplasm
YGL208W	YGL115W_2	8.48E-57	
YDR422C	YGL115W_2	3.64E-37	cytoplasm
YER027C	YGL115W_3	6.83E-77	cytoplasm
YGL208W	YGL115W_3	6.37E-71	
YDR028C	YGL115W_3	9.81E-38	cytoplasm
YER027C	YGL115W_4	5.62E-22	cytoplasm
YGL208W	YGL115W_4	7.23E-24	
YDR477W	YGL115W_4	1.89E-14	cytoplasm

Predictions of subcellular localization were based on the known localizations of 4156 budding yeast proteins [30], where there are 22 categories of subcellular location. Predictive accuracy was evaluated by comparing predicted locations to the known locations of these proteins as reported in the ontology annotation of the Saccharomyces Genome Database (SGD, ).

### Homology modeling

NCBI's online BLAST engine  was used to search PDB database for protein sequences similar to the selected yeast protein sequences. The best match was selected as a template structure and its PDB file was downloaded from the PDB database. All homology modeling was carried out with MOE (Molecular Operating Environment 2004.03, The Chemical Computing Group Inc., 2004). The query sequences and their templates were first aligned in MOE. Ten intermediate models were then created, each was finely energy-minimized for steric interactions using the AMBER-94 forcefield with the solvation option turned on. The best structure prediction was then selected according to energy ranking.

## Authors' contributions

JWF designed the project. JWF and RJH carried out the study and drafted the manuscript. YHD and GHL participated in the study and manuscript preparation.

## Supplementary Material

Additional File 1 Homology models of five yeast proteins. The following files are available in the complementary website : MEME output files for all novel signatures, PDB files of five homology models, a complete list of identified novel signatures and a list of these signatures grouped by similarity, a complete list of protein location prediction, and the distribution of the number of interaction partners. Click here for file
