# Supplementary material for: Discover protein sequence signatures from protein-protein interaction data
Source: BMC Bioinformatics. 2005 Nov 23;6:277. doi: 10.1186/1471-2105-6-277 (PMC1310605; doi:10.1186/1471-2105-6-277)
Supplement: Additional File 1 — Homology models of five yeast proteins. The following files are available in the complementary website : MEME output files for all novel signatures, PDB files of five homology models, a complete list of identified novel signatures and a list of these signatures grouped by similarity, a complete list of protein location prediction, and the distribution of the number of interaction partners. [file 1471-2105-6-277-S1.doc]

**Additional file 1**. Homology models of yeast proteins. Sequence signatures were highlighted in red. *P*-values in Identity/Similarity column are BLAST *p*-values against PDB database. The underscore notation in the signature column indicates which signatures in the set of guest proteins of the named host. The solvent accessibility column displays the ratio of exposed residues of signatures and the whole proteins (in parentheses).

| **Yeast Protein** | **Template Structure** | **Identity/Similarity** | **Signature** | **Solvent accessibility** | **Homology Model** |
| --- | --- | --- | --- | --- | --- |
| YLR180W | 1O93 | %Alignment = 99.2%  *P*-value = 6.7e-124  %Identity = 69  %Similar = 18 | YDL201W_4 | 7/11 (199/380) | 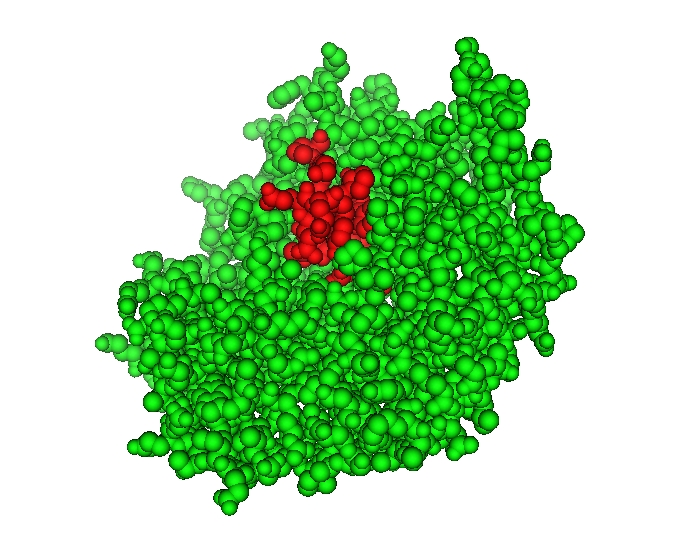 |
| YJR047C | 1X6O | %Alignment = 96.8%  *P*-value = 4.4e-36  %Identity = 51  %Similar = 26 | YHR046C_5 | 12/14 (104/141) | 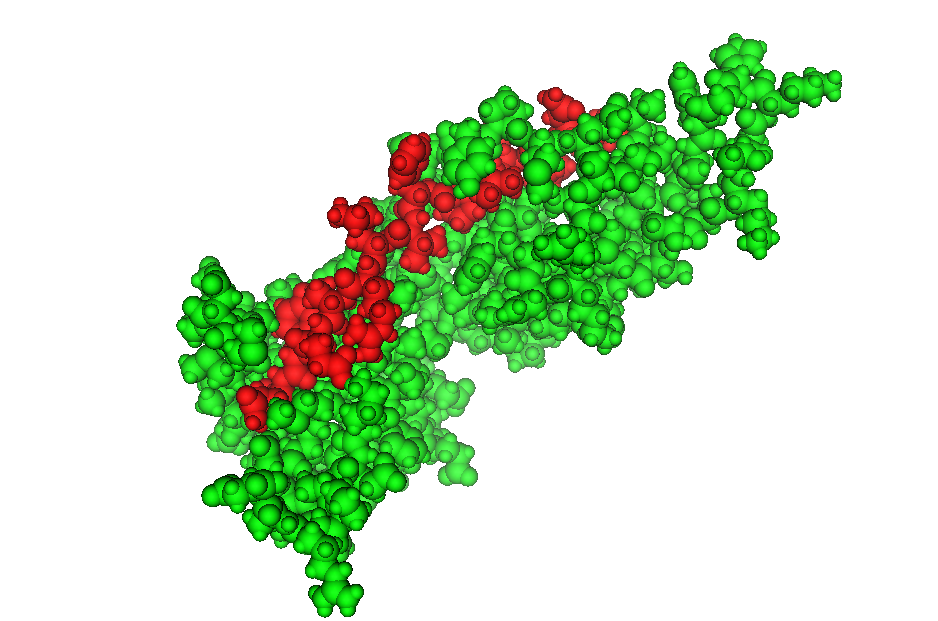 |
| YOR111W | [1EX2](http://db.yeastgenome.org/cgi-bin/protein/get3d?locus=YOR111W&pdb=1ex2_B&align=1#structure) | %Alignment = 88%  *P*-value = 2.0e-13  %Identity = 32  %Similar = 31 | YOR335C_5 | 20/26 (145/217) | 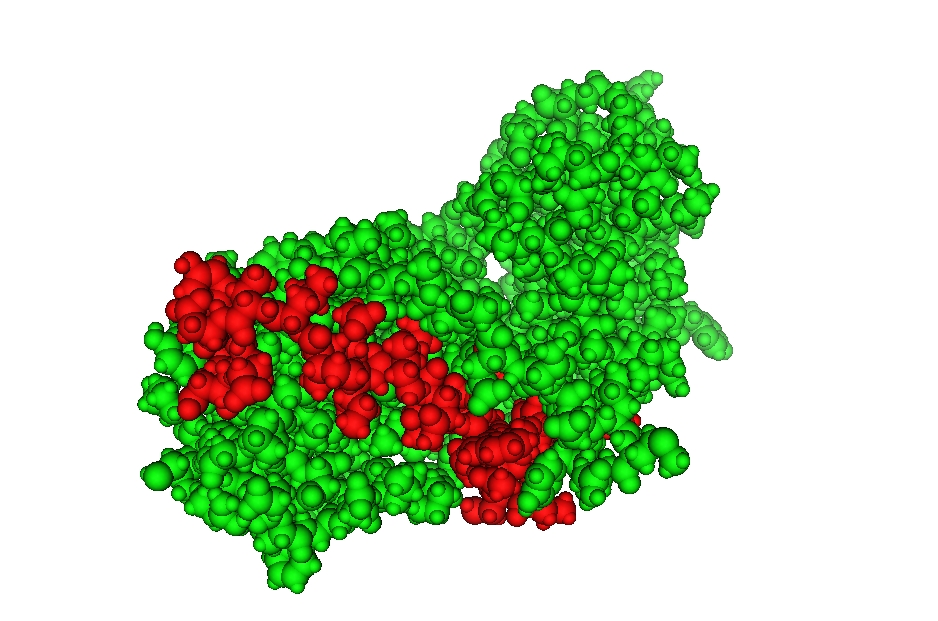 |
| YKL113C | 1UL1 | %Alignment = 97.9%  *P*-value = 2.1e-98  %Identity = 58  %Similar = 23 | YNL250W_1 | 15/21 (219/343) | 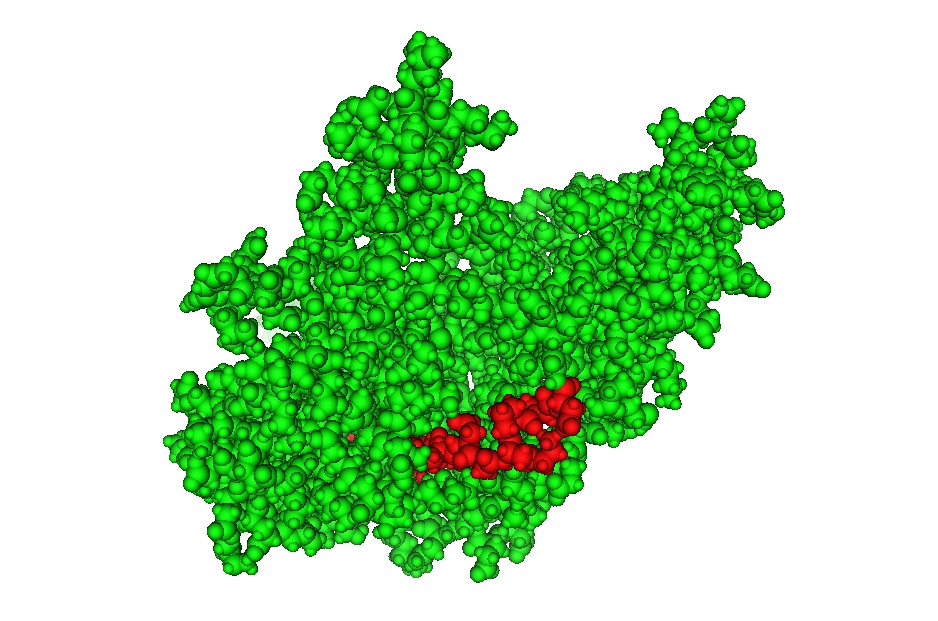 |
| YGL205W | 1LS2 | %Alignment = 95.2%  *P*-value = 1.0e-64  %Identity = 29  %Similar = 33 | YKL211C_5 | 8/14 (398/689) | 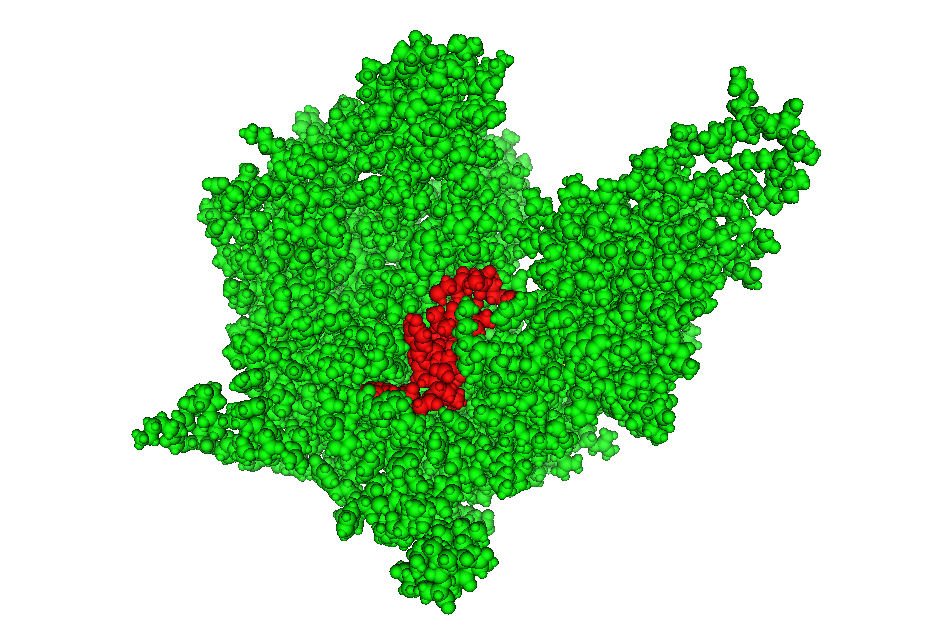 |
